# Supplementary material for: The FlagT4G Vaccine Confers a Strong and Regulated Immunity and Early Virological Protection against Classical Swine Fever
Source: Viruses. 2022 Sep 2;14(9):1954. doi: 10.3390/v14091954 (PMC9502879; doi:10.3390/v14091954)

Supplementary data S1. Flow cytometry gating strategy

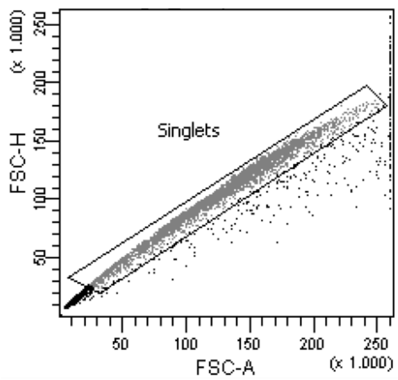

First gate: separating singlets from doublets

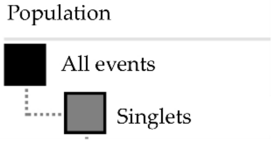

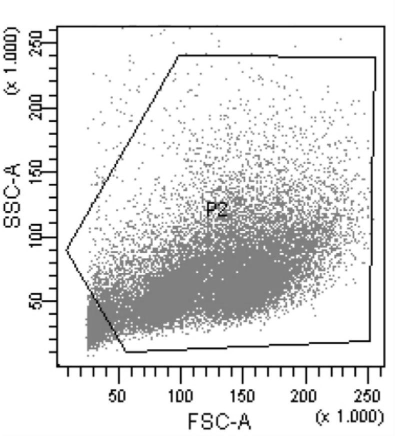

Second gate: Population discrimination according to size and complexity

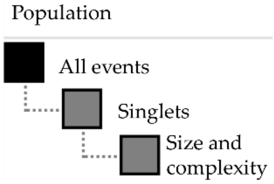

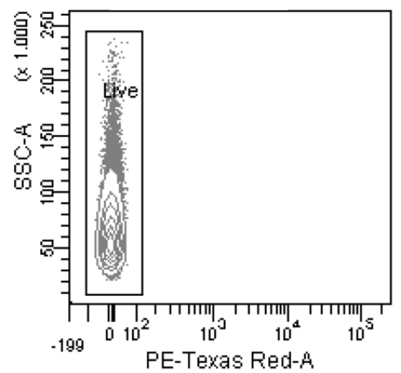

Third gate: Unstained cells used to stablish the threshold for the viability staining (detected on the PE-Texas red channel). Live cells will remain unstained.

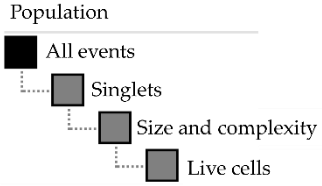

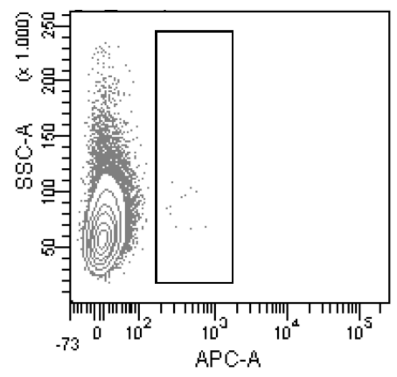

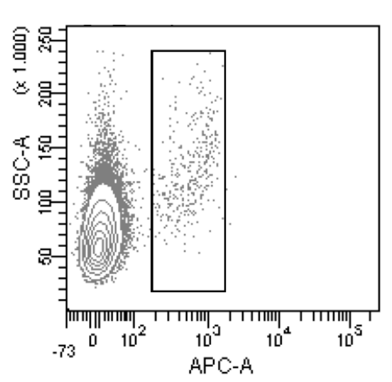

Fourth gate: Used exclusively for the CD172+ staining (IgG1 hybridome supernatant), which uses goat anti-mouse IgG1, Alexa-647 labelled, as a secondary antibody. Cells are labelled solely with the secondary antibody, to establish the background fluorescence from the fluorochrome (detected on the APC channel). This background will be taken into account to gate the positive cells in the real sample (which will include primary and secondary staining).

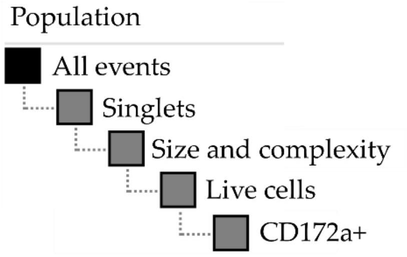

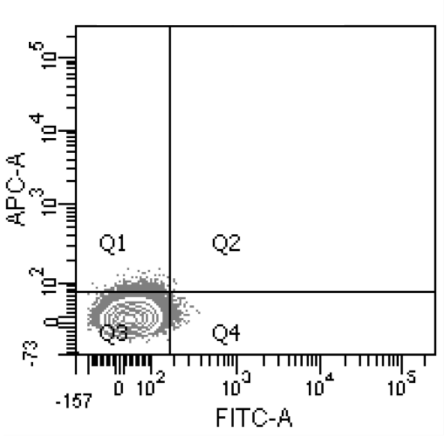

Fifth gate: Used for the CD4/CD8 staining.

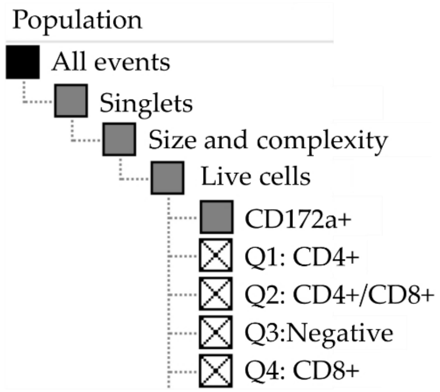

Supplement: Supplementary file 1 [file viruses-14-01954-s001.zip › Supplementary data S1.pdf]
