# Supplementary material for: The FlagT4G Vaccine Confers a Strong and Regulated Immunity and Early Virological Protection against Classical Swine Fever
Source: Viruses. 2022 Sep 2;14(9):1954. doi: 10.3390/v14091954 (PMC9502879; doi:10.3390/v14091954)
Supplement: Supplementary file 1 [file viruses-14-01954-s001.zip › Table S1.pdf]

Table S1. Cytokine (IFN-α, IFN-γ, IL-1β, IL-4, IL-6, IL-8, IL-10, IL-12 and TNF-α) individual response after CSFV vaccination and challenge. Cytokines were quantified on a Luminex® 200™ (pg/mL). All the samples were negative for IFN-γ levels in sera at all the evaluated time points.
Group A (FlagT4G vaccinated): Pigs 1-5. Group B (Unvaccinated): Pigs 6-10.

	Pig Nº	Day of vaccination	Day of CSFV Challenge	4 DPC	7 DPC
**IFNα**	**1**	3.96	203.6	2.01	0
	**2**	34.39	202.43	4.36	8.66
	**3**	39.63	130.83	8.37	6.89
	**4**	6.81	166.04	3.51	1.97
	**5**	24.35	148.65	4.44	2.42
	**6**	3.55	0	209.62	24.38
	**7**	2.42	0	172.09	89.89
	**8**	1.05	0	85.67	143.75
	**9**	10.95	1.21	89.78	58.32
	**10**	9.45	0.97	281.78	64.31
**IL-1β**	**1**	0	76.68	1211.65	1778.92
	**2**	2449.65	994.38	23.5	0
	**3**	98.94	1161.84	654.28	203.2
	**4**	418.97	180.72	111.03	316.81
	**5**	1183.47	139.4	194.24	0
	**6**	299.59	81.69	0	0
	**7**	277.96	11.87	0	0
	**8**	0	0	0	0
	**9**	29.15	23.5	45.59	0
	**10**	1095.03	91.6	148.69	76.68

	Pig Nº	Day of vaccination	Day of CSFV Challenge	4 DPC	7 DPC
**TNFα**	**1**	0	0	138.36	191.44
	**2**	323.79	169.36	0	0
	**3**	0	21.42	0	0
	**4**	57	6.87	0	0
	**5**	317.59	0	0	0
	**6**	0	0	0	0
	**7**	95.97	0	0	0
	**8**	0	0	0	0
	**9**	0	0	21.42	0
	**10**	77.27	0	0	0
**IL-4**	**1**	0	0	7.24	23.3
	**2**	40.8	36.48	0	0
	**3**	0	22.27	18.08	0
	**4**	16.48	9.85	0.81	8.7
	**5**	29.37	2.06	0	0
	**6**	0	0	1.43	0
	**7**	2.69	0	0	0
	**8**	0	0	0	0
	**9**	0	0	3.31	0
	**10**	10.42	0	5.75	2.06

	Pig Nº	Day of vaccination	Day of CSFV Challenge	4 DPC	7 DPC
**IL-6**	**1**	0	0	0	0
	**2**	3.83	28.6	0	0
	**3**	0	0	0	0
	**4**	0	0	0	0
	**5**	0	0	0	0
	**6**	66.12	11.43	68.41	6.38
	**7**	0	0	0	0
	**8**	6.38	0	0	6.38
	**9**	0	0	6.38	0
	**10**	18.87	3.83	33.4	0
**IL-8**	**1**	0	65.43	49.13	53.02
	**2**	200.04	157.99	69.19	0
	**3**	189.45	367.28	239.73	0
	**4**	24.77	13.8	0	0
	**5**	371.81	205.71	111.71	55.91
	**6**	58.31	129.11	117.4	0
	**7**	24.77	34.14	11.51	0
	**8**	0	71.99	118.71	205.71
	**9**	30.02	0	49.13	0
	**10**	24.77	0	0	0

	Pig Nº	Day of vaccination	Day of CSFV Challenge	4 DPC	7 DPC
**IL-12**	**1**	103.38	560.36	2615.33	3049.4
	**2**	3077.13	2917.07	577.46	190.87
	**3**	358.35	1179.54	1054.45	298.34
	**4**	255.5	284.2	185.72	848.55
	**5**	923.45	590.25	443.28	180.53
	**6**	695.97	226.15	362.89	0
	**7**	921.37	218.69	385.47	0
	**8**	719.06	403.38	1187.89	1181.63
	**9**	265.13	65.83	1461.7	154.08
	**10**	392.2	211.18	840.22	330.89
**IL-10**	**1**	0	0	0	0
	**2**	0	33.91	0	0
	**3**	21.87	0	0	0
	**4**	0	0	0	0
	**5**	0	0	0	16.87
	**6**	0	0	38.51	49.51
	**7**	0	0	0	0
	**8**	0	0	0	26.78
	**9**	0	0	91.25	0
	**10**	0	0	0	0
